# Are We There Yet? The Smallpox Research Agenda Using Variola Virus

**DOI:** 10.1371/journal.ppat.1004108

**Published:** 2014-05-01

**Authors:** Inger K. Damon, Clarissa R. Damaso, Grant McFadden

**Affiliations:** 1 Poxvirus and Rabies Branch, Division of High Consequence Pathogens and Pathology, National Center for Emerging and Zoonotic Diseases, Centers for Disease Control and Prevention, Atlanta, Georgia, United States of America; 2 Instituto de Biofisica Carlos Chagas Filho, Universidade Federal do Rio de Janeiro, Rio de Janeiro, Rio de Janeiro, Brazil; 3 Department of Molecular Genetics and Microbiology, College of Medicine, University of Florida, Gainesville, Florida, United States of America; The Fox Chase Cancer Center, United States of America

Despite significant advances, there is more work to be done before the international community can be confident that it possesses sufficient protection against any future smallpox threats. The current World Health Organization (WHO)-approved research agenda for smallpox has been tightly focused by the interpretation that research “essential for public health” equates solely to applied research related directly to the development of new antiviral drugs, safer vaccines, and better diagnostics. Despite considerable advances in this direction, we argue that the research agenda with live variola virus is not yet finished and that significant gaps still remain.

Variola virus is unique amongst the orthopoxviruses in that it is known to be a sole human pathogen. The viral and host factors responsible for this human-specific tropism remain essentially unknown to this day, although the current genomic information across orthopoxviruses makes hypothesis-driven experimental design using functional genomic approaches more feasible. Indeed, greater exploitation of current technologies may lead to additional therapeutic or diagnostic products to better respond to any future emergency situation resulting from a smallpox appearance.

Smallpox disease was declared eradicated in 1980, and today is the only human disease to be eliminated by WHO. Shortly after WHO officially declared smallpox eradicated, a decision was made to ultimately destroy the remaining stocks of live variola virus, with interim use of the virus permitted only for defined WHO-approved research projects. Variola virus stocks were then voluntarily consolidated in the early 1980s to two WHO Collaborating Center laboratories, one in Russia and the other in the United States, which remain the only two WHO-approved sites for research with live variola virus.

In 1999, following an independent report on variola research by the Institute of Medicine (IOM), a decision by WHO was made to increase the amount of research that utilized live variola virus. The WHO Advisory Committee on Variola Virus Research (ACVVR) was subsequently formed to oversee the research, and research began to generate additional virus genomic information, characterize new antivirals and therapeutics, evaluate newer generations of vaccines and biologics, develop diagnostics, better define disease pathogenesis, and generate animal models of smallpox disease. This work was further refined by the World Health Assembly (WHA) to focus on “essential public health research” in 2005 and was subsequently comprehensively reviewed by the IOM in 2009 [1], and the WHO ACVVR and its assembled external advisory group (called AGIES) in 2010 and 2013. Each of the last two comprehensive reviews was prepared to summarize research advances and to recommend whether additional research with live virus would be required in order to fulfill the original WHO-mandated agenda, in advance of a WHA-wide discussion about the fate of the remaining variola virus materials stored at the two WHO Collaborating Centers. These reviews are available online (http://apps.who.int/iris/bitstream/10665/97033/1/WHO_HSE_PED_CED_2013.2_eng.pdf; http://apps.who.int/iris/bitstream/10665/97034/1/WHO_HSE_PED_CED_2013.3_eng.pdf). Because of the advances made in the acquisition of knowledge to support diagnostics, antiviral, and vaccine research and development through to the regulatory review process, the majority opinions of those in these groups are now, in 2014, more supportive of discontinuing the use of live variola virus for future research studies.

Significant advances in our abilities to diagnose, treat, and to prevent smallpox disease have been made in the past two decades, largely as a function of concerted efforts using surrogate orthopoxviruses, and work with live variola virus that has, up to now, been accepted as needed under the ACVVR framework. Diagnostic advances have been used to rule out suspected cases of smallpox [2] and to diagnose other related orthopoxvirus diseases, such as the cases of human monkeypox in the United States in 2003 [3], and enhancements of surveillance for monkeypox in the Democratic Republic of Congo [4]. As a result, improved recognition of smallpox-like diseases has been greatly augmented. Increased recognition and characterization of enzootic human infections with vaccinia [5] and cowpox [6] have, respectively, also been made in South America and Eurasia [7]. Rapid and specific nucleic acid–based tests for more accurate diagnosis of smallpox, as well as other orthopoxvirus-related diseases, have been published in the peer-reviewed literature. A real-time PCR-based assay system has received regulatory approval in Russia, and an orthopoxvirus (non-variola) test has met regulatory review standards for use in the Laboratory Response Network (LRN) in the US [2]. Work continues to develop protein-based diagnostic assays, which can allow more rapid “alerts” to any cases of possible smallpox disease. Because the latter assays are often done simply, similar to a urine pregnancy test, these could be critical to target attention to any potential areas of high-risk disease. However, more efforts to finalize a dependable and reproducible product for clinical use are not yet completed, and key validation tests still need to be conducted.

Several new antiviral drug candidates have now been developed and shown to have benefit in the treatment of orthopoxvirus disease, including variola infection of nonhuman primates [8]. Two antivirals with different mechanisms of action are now in advanced stages of development as potential smallpox drugs. ST-246, also known as Tecovirimat or Arestyvir, is a virion egress inhibitor with specificity for orthopoxviruses [9], and CMX001, also called Brincidofovir, is a DNA polymerase inhibitor active against multiple DNA viruses and derivative of a licensed antiviral drug called cidofovir [10]; both are orally bioavailable. The former is now stockpiled in the US strategic national stockpile after advanced development, but is not yet licensed by the Food and Drug Administration (FDA); its use for treatment of orthopoxvirus infections, including smallpox, is regulated via an investigational new drug (IND) process operated by the Centers for Disease Control and Prevention (CDC), and hopefully will be transitioned to a more streamlined emergency use authorization (EUA) process. Limited studies with ST-246 have been performed to show protection against death and decreased viral shedding when variola is the challenge virus in nonhuman primates [11]. Off-label use of cidofovir and investigational use of Brincidofovir and Arestyvir, in addition to vaccinia immune globulin (VIG), have been successfully used in treatment of a severe case of eczema vaccinatum and of progressive vaccinia in humans [12], [13]. Continued investigation to identify additional candidate antismallpox drugs, for example, to screen currently approved kinase inhibitors, may provide additional antiorthopoxvirus and antismallpox therapeutics.

The original vaccines used successfully to eradicate smallpox in the 1960s and 70s are now in limited supply and, furthermore, were associated with what we now consider to be an unacceptably high rate of adverse events, some severe [14]. In their stead, cell culture–derived smallpox vaccines have been developed, some derived from clonal derivatives of the historic vaccine strains [15]. Additionally, less reactogenic smallpox vaccines, such as IMVAMUNE and Lc16m8 [16], [17], [18], [19], are now in advanced development. IMVAMUNE recently met the European Medicines Agency (EMA) standards for use for prevention of smallpox, and Lc16m8 has been licensed for use in Japan. Live variola virus has been used as a target for neutralization assays by vaccinee sera [20], as a surrogate to understand how the immune response elicited by these vaccines compares with the historic vaccines or the newer versions of vaccines directly derived from the historic vaccines. As orthopoxvirus infections are reemerging, particularly monkeypox and cowpox [3], [5], [6], [21], these vaccines are tools not just to be used for a smallpox response but that can also be used for orthopoxvirus disease prevention in general.

Despite these advances, we argue that there is more to be done. While certain aspects of the original research goals using live virus have been met, other key items, like the wider approval of accurate diagnostics that can distinguish smallpox from other orthopoxvirus diseases or the full licensure of new antiviral drugs and vaccines that are effective against variola virus, have not yet been completed.

Even in the past ten years, molecular technologies have advanced considerably. The continued use of variola virus (or its genomic material) may be needed to evaluate how well newer generations of diagnostics, for instance, will perform in the newer diagnostic platforms. Current generations of DNA sequencing technologies are now far advanced compared to those of 1999 and may soon supplant PCR-based diagnostics in some advanced laboratories. But these laboratories will not be able to handle all smallpox diagnostic capacity in a timely fashion; the reliance on many international microbiology laboratories will be necessary in the event of any reemergence of smallpox in the future, and protein-based diagnostic assays will augment laboratory-based surveillance and detection strategies. Although two antiviral drugs with different mechanisms of action are in advanced stages of development, resistance to each of these drugs has been elicited in cultured cells and against one of these drugs in a vaccinia-infected human [9], [13], [22], [23]. Similarly, the demonstration more than a decade ago of a recombinant ectromelia (mousepox) virus construct that expresses IL-4 and is more resistant to the smallpox vaccine [24] has raised concerns of the potential creation of a vaccine-resistant smallpox virus. While the likelihood of the emergence of, or creation of, either drug- or vaccine-resistant versions of smallpox is unknown, continued investigation to identify additional countermeasures, for example, through screening using functional genomics or proteomics approaches, can further enhance our state of preparedness. Additional studies evaluating the safety and efficacy of drug combination therapies will also be needed. Certainly the current capabilities of synthetic biology and the availability of multiple variola virus genome sequences in the published literature make these scenarios more worrisome in the 21st century and also make the feasibility of ultimate final destruction of variola virus, itself, problematic [25].

Despite this changing landscape, the WHO-approved research agenda has largely become conscribed to the needs of finalizing the work on the remaining antiviral product issues. Fundamental research has been greatly limited over the past decade. Thus, basic variola virus research projects that could potentially lead to other advances in public health efforts have become increasingly absent from the list of WHO-approved projects. It should be noted that the international scientific community has fully complied with these WHO strictures for conducting work with live variola virus. Also, input from various external bodies, such as the Institute of Medicine, has been received and considered by the WHO in order to develop a coherent research agenda for live variola virus. Unique amongst orthopoxviruses, which are largely zoonotic pathogens, variola is known to be a sole human pathogen. The viral and host factors responsible for this specific tropism remain essentially unknown, although the current genomic information database across orthopoxviruses makes hypothesis-driven experimental design using functional genomic approaches more feasible than in the past. We recognize that ultimate proof of such hypotheses will be challenging, as current animal models using variola virus do not faithfully recapitulate the human clinical disease process or immune responses [26], and recombinant genetic modification approaches are not condoned in use of variola. We recommend that the scientific and world community re-engage to discuss future research potential with live variola virus to improve disease interventions by advancing our understanding of the virus and its relationship with its human host.

In May 2014, the WHA will consider whether to destroy the remaining stocks of live variola virus or, instead, to recommend continued research with live variola virus in the two WHO-certified sites. This research remains vital, and we believe that the original goals of the WHO agenda for newer and safer vaccines, fully licensed antiviral drugs, and better diagnostics have still not been fully met.

## References

[1] Institute of Medicine (2009) Live Variola Virus: Considerations for Continuing Research. Washington (D.C.): The National Academies Press. Available: http://www.iom.edu/Reports/2009/LiveVariolaVirusContinuingResearch.aspx. Accessed: 26 March 2014.

[2] WHO (2010) Scientific review of variola virus research, 1999–2010. Available: http://www.who.int/csr/disease/smallpox/major_review/en/. Accessed: 26 March 2014

[3] Reed KD, Melski JW, Graham MB, Regnery RL, Sotir MJ, et al (2004) The detection of monkeypox in humans in the Western Hemisphere. N Engl J Med 350: 342–350. 14736926 10.1056/NEJMoa032299

[4] Li Y, Olson VA, Laue T, Laker MT, Damon IK (2006) Detection of monkeypox virus with real-time PCR assays. J Clin Virol 36: 194–203. 16731033 10.1016/j.jcv.2006.03.012 PMC9628957

[5] Quixabeira-Santos JC, Medaglia ML, Pescador CA, Damaso CR (2011) Animal movement and establishment of vaccinia virus cantagalo strain in Amazon biome, Brazil. Emerg Infect Dis 17: 726–729. 21470472 10.3201/eid1704.101581 PMC3377419

[6] Dabrowski PW, Radonic A, Kurth A, Nitsche A (2013) Genome-wide comparison of cowpox viruses reveals a new clade related to variola virus. PLoS One 8: e79953. 24312452 10.1371/journal.pone.0079953 PMC3848979

[7] Moussatche N, Damaso CR, McFadden G (2008) When good vaccines go wild: Feral Orthopoxvirus in developing countries and beyond. J Infect Dev Ctries 2: 156–173. 19738346 10.3855/jidc.258

[8] Prichard MN, Kern ER (2012) Orthopoxvirus targets for the development of new antiviral agents. Antiviral Res 94: 111–125. 22406470 10.1016/j.antiviral.2012.02.012 PMC3773844

[9] Yang G, Pevear DC, Davies MH, Collett MS, Bailey T, et al (2005) An orally bioavailable antipoxvirus compound (ST-246) inhibits extracellular virus formation and protects mice from lethal orthopoxvirus Challenge. J Virol 79: 13139–13149. 16189015 10.1128/JVI.79.20.13139-13149.2005 PMC1235851

[10] Parker S, Touchette E, Oberle C, Almond M, Robertson A, et al (2008) Efficacy of therapeutic intervention with an oral ether-lipid analogue of cidofovir (CMX001) in a lethal mousepox model. Antiviral Res 77: 39–49. 17904231 10.1016/j.antiviral.2007.08.003 PMC9628989

[11] Mucker EM, Goff AJ, Shamblin JD, Grosenbach DW, Damon IK, et al (2013) Efficacy of tecovirimat (ST-246) in nonhuman primates infected with variola virus (Smallpox). Antimicrob Agents Chemother 57: 6246–6253. 24100494 10.1128/AAC.00977-13 PMC3837858

[12] Vora S, Damon I, Fulginiti V, Weber SG, Kahana M, et al (2008) Severe eczema vaccinatum in a household contact of a smallpox vaccinee. Clin Infect Dis 46: 1555–1561. 18419490 10.1086/587668

[13] Lederman ER, Davidson W, Groff HL, Smith SK, Warkentien T, et al (2012) Progressive vaccinia: case description and laboratory-guided therapy with vaccinia immune globulin, ST-246, and CMX001. J Infect Dis 206: 1372–1385. 22904336 10.1093/infdis/jis510 PMC3529603

[14] Kretzschmar M, Wallinga J, Teunis P, Xing S, Mikolajczyk R (2006) Frequency of adverse events after vaccination with different vaccinia strains. PLoS Med 3: e272. 16933957 10.1371/journal.pmed.0030272 PMC1551910

[15] Monath TP, Caldwell JR, Mundt W, Fusco J, Johnson CS, et al (2004) ACAM2000 clonal Vero cell culture vaccinia virus (New York City Board of Health strain)–a second-generation smallpox vaccine for biological defense. Int J Infect Dis 8 Suppl 2: S31–44. 15491873 10.1016/j.ijid.2004.09.002 PMC7110559

[16] Kennedy JS, Gurwith M, Dekker CL, Frey SE, Edwards KM, et al (2011) Safety and immunogenicity of LC16m8, an attenuated smallpox vaccine in vaccinia-naive adults. J Infect Dis 204: 1395–1402. 21921208 10.1093/infdis/jir527 PMC3218648

[17] Greenberg RN, Overton ET, Haas DW, Frank I, Goldman M, et al (2013) Safety, immunogenicity, and surrogate markers of clinical efficacy for modified vaccinia Ankara as a smallpox vaccine in HIV-infected subjects. J Infect Dis 207: 749–758. 23225902 10.1093/infdis/jis753 PMC3611764

[18] Frey SE, Winokur PL, Salata RA, El-Kamary SS, Turley CB, et al (2013) Safety and immunogenicity of IMVAMUNE(R) smallpox vaccine using different strategies for a post event scenario. Vaccine 31: 3025–3033. 23664987 10.1016/j.vaccine.2013.04.050 PMC3755481

[19] Kennedy RB, Ovsyannikova I, Poland GA (2009) Smallpox vaccines for biodefense. Vaccine 27 Suppl 4: D73–79. 19837292 10.1016/j.vaccine.2009.07.103 PMC2764553

[20] Damon IK, Davidson WB, Hughes CM, Olson VA, Smith SK, et al (2009) Evaluation of smallpox vaccines using variola neutralization. J Gen Virol 90: 1962–1966. 19339477 10.1099/vir.0.010553-0 PMC2887573

[21] Shchelkunov SN (2013) An increasing danger of zoonotic orthopoxvirus infections. PLoS Pathog 9: e1003756. 24339772 10.1371/journal.ppat.1003756 PMC3855571

[22] Smee DF, Sidwell RW, Kefauver D, Bray M, Huggins JW (2002) Characterization of wild-type and cidofovir-resistant strains of camelpox, cowpox, monkeypox, and vaccinia viruses. Antimicrob Agents Chemother 46: 1329–1335. 11959564 10.1128/AAC.46.5.1329-1335.2002 PMC127179

[23] Smee DF, Wandersee MK, Bailey KW, Hostetler KY, Holy A, et al (2005) Characterization and treatment of cidofovir-resistant vaccinia (WR strain) virus infections in cell culture and in mice. Antivir Chem Chemother 16: 203–211. 16004083 10.1177/095632020501600306

[24] Jackson RJ, Ramsay AJ, Christensen CD, Beaton S, Hall DF, et al (2001) Expression of mouse interleukin-4 by a recombinant ectromelia virus suppresses cytolytic lymphocyte responses and overcomes genetic resistance to mousepox. J Virol 75: 1205–1210. 11152493 10.1128/JVI.75.3.1205-1210.2001 PMC114026

[25] McFadden G (2010) Killing a killer: what next for smallpox? PLoS Pathog 6: e1000727. 20126444 10.1371/journal.ppat.1000727 PMC2813274

[26] Cann JA, Jahrling PB, Hensley LE, Wahl-Jensen V (2013) Comparative pathology of smallpox and monkeypox in man and macaques. J Comp Pathol 148: 6–21. 22884034 10.1016/j.jcpa.2012.06.007 PMC3498598

